# Supplementary material for: 3p Arm Loss and Survival in Head and Neck Cancer: An Analysis of TCGA Dataset
Source: Cancers (Basel). 2021 Oct 22;13(21):5313. doi: 10.3390/cancers13215313 (PMC8582539; doi:10.3390/cancers13215313)
Supplement: Supplementary file 1 [file cancers-13-05313-s001.zip › Supplementary Material 3pdel.pdf]

Supplementary Material

Supplementary Figure S1. Homozygous deletion on the 3p arm is a rare event.
Supplementary Figure S2. Concordance between cohorts with 3p14.2 fragile site loss and 97% 3p arm loss in HPV-negative samples. In the HPV-negative cohort, patients with 3p14.2 fragile site loss almost universally had loss of 97% of the 3p arm (Fisher test $p<10^{-64}$).
Supplementary Figure S3. Overall survival by 3p deletion status in the MSK-IMPACT cohort. A) Full 3p loss in HPV-negative samples, B) full 3p loss in HPV-positive samples, C) any 3p loss in HPV-negative samples, D) any 3p loss in HPV-positive samples. Log-rank tests were used for survival comparisons.
Supplementary Figure S4. Comparison of A) mutation load and B) percent genome altered by 3p arm status and HPV status. Mann-Whitney Wilcoxon tests were used to make the comparisons, and the resulting *p* values are shown.
Supplementary Figure S5. Visualization of CNA events across all genes and tumor samples in our HPV-negative cohorts. Every gain and deletion as termed by GISTIC2's CNA analysis in the matrix of 24,776 genes × 375 tumor samples was colored red or blue, respectively.
Supplementary Figure S6. Correlation of CNA and mRNA abundance results between by 3p arm status in HPV-negative tumors. A) Deletions and B) Amplifications. Log (base 10) ranked signed FDR values of CNA and mRNA abundance are mapped on the x- and y-axis, respectively. Colored dots represent genes with at least a two-fold change in mRNA abundance. Genes with higher copy number (FDR<0.1) and mRNA abundance (FDR<0.01) in 3p arm loss are colored red, while genes with lower copy number (FDR<0.1) and mRNA abundance (FDR<0.01) in 3p arm loss are colored blue. Genes on the 3p arm are not shown.
Supplementary Table S1. Demographic differences by 3p arm status in MSK-IMPACT HNSCC cohort HPV-negative samples.
Supplementary Table S2. Demographic differences by 3p arm status in MSK-IMPACT HNSCC cohort HPV-positive samples.
Supplementary Table S3. Demographic differences by fragile site status in all samples.
Supplementary Table S4. Multivariate analysis of overall survival in the HPV-negative cohort by 3p arm status. The multivariate model was constructed through backwards stepwise analysis.
Supplementary Tables S5-8: see Excel file
Supplementary Table S9. Pathways significantly overrepresented with SNV and integrated CNA-mRNA differences between 3p arm status. Genes with higher copy number and higher mRNA abundance in 3p arm loss are colored red, genes with lower copy number and lower mRNA abundance in are colored blue, and genes with differences in SNV are uncolored.
Supplementary Table S10. TME comparisons by 3p arm status. Scores were computed using the MCP-counter method, normalized using Box-Cox transformation, and compared using linear regression. FDR correction was done using Benjamini-Hochberg method. Significant values are bolded.
Supplementary Table S11. Comparison of hypoxia profiles by 3p arm status. All eight hypoxia scores in Bhandari *et al.*'s supplementary materials were normalized using Box-Cox transformation and compared using linear regression [17]. FDR correction was done using Benjamini-Hochberg method. All values were significant.
Supplementary Tables S12-13: Please see excel file.

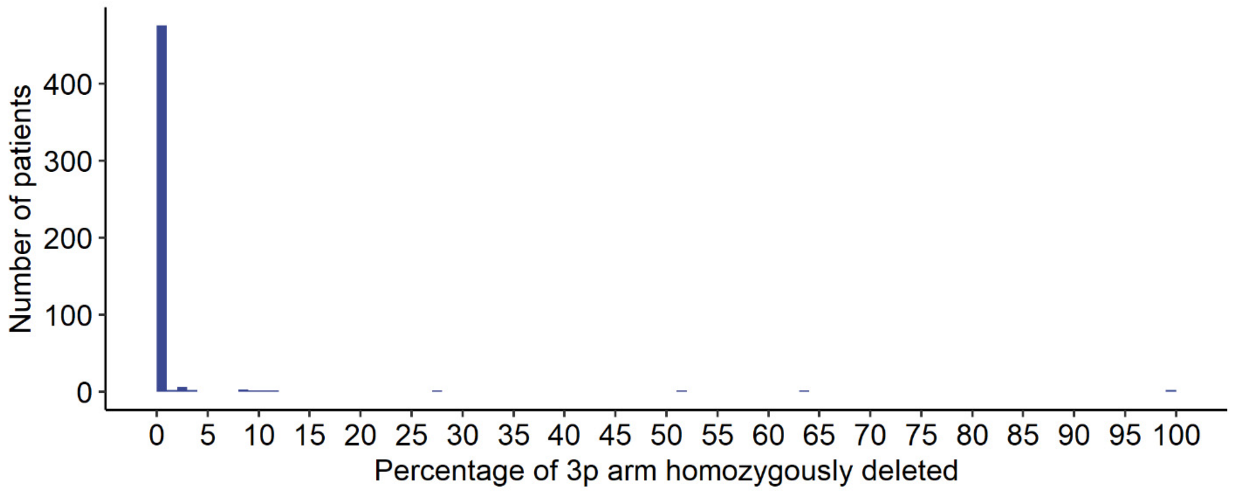

Supplementary Figure S1. Homozygous deletion on the 3p arm is a rare event.

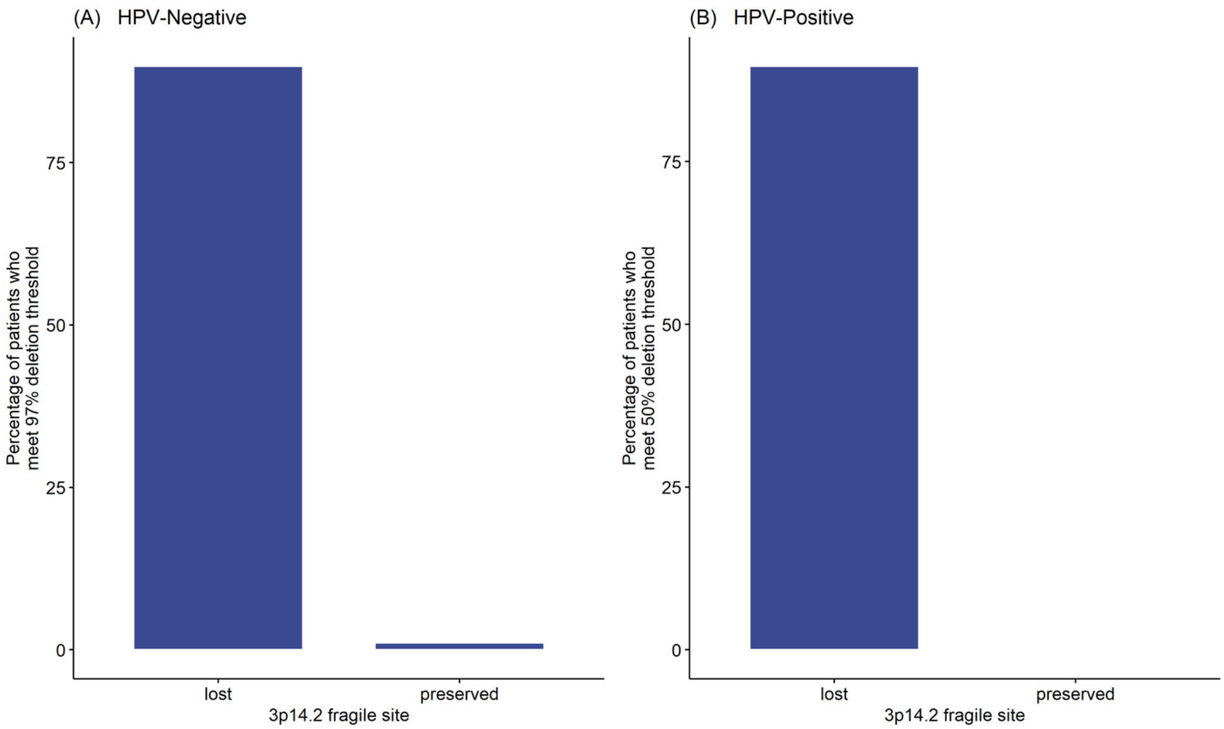

Supplementary Figure S2. Concordance between cohorts with 3p14.2 fragile site loss and 97% 3p arm loss in A) HPV-negative and B) HPV-positive samples. In each cohort, patients with 3p14.2 fragile site loss almost universally had loss of 97% and 50% of the 3p arm, respectively (Fisher test $p<10^{-64}$ and $p<10^{-16}$, respectively).

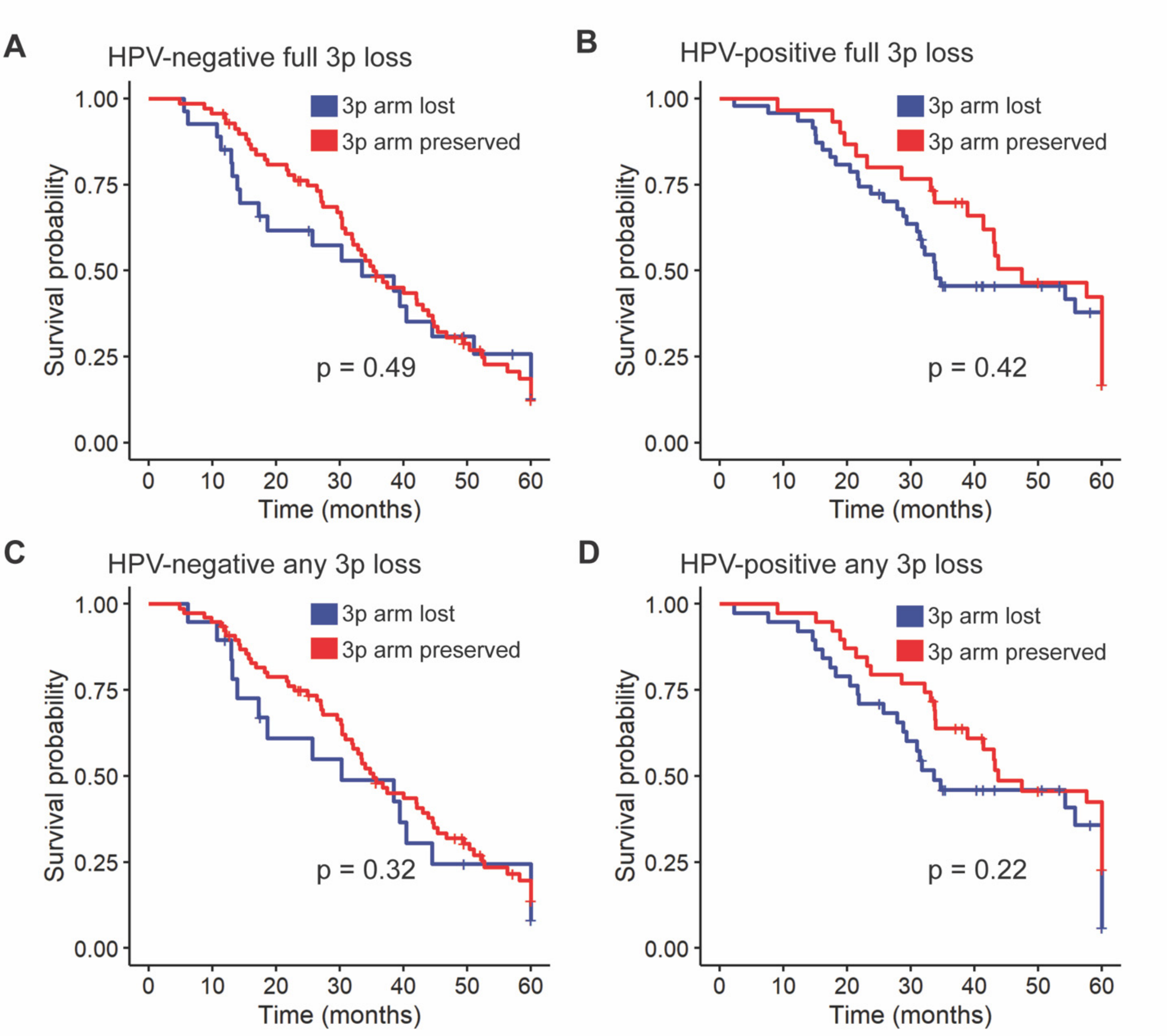

Supplementary Figure S3. Overall survival by 3p deletion status in the MSK-IMPACT cohort. A) Full 3p loss in HPV-negative samples, B) full 3p loss in HPV-positive samples, C) any 3p loss in HPV-negative samples, D) any 3p loss in HPV-positive samples. Log-rank tests were used for survival comparisons.

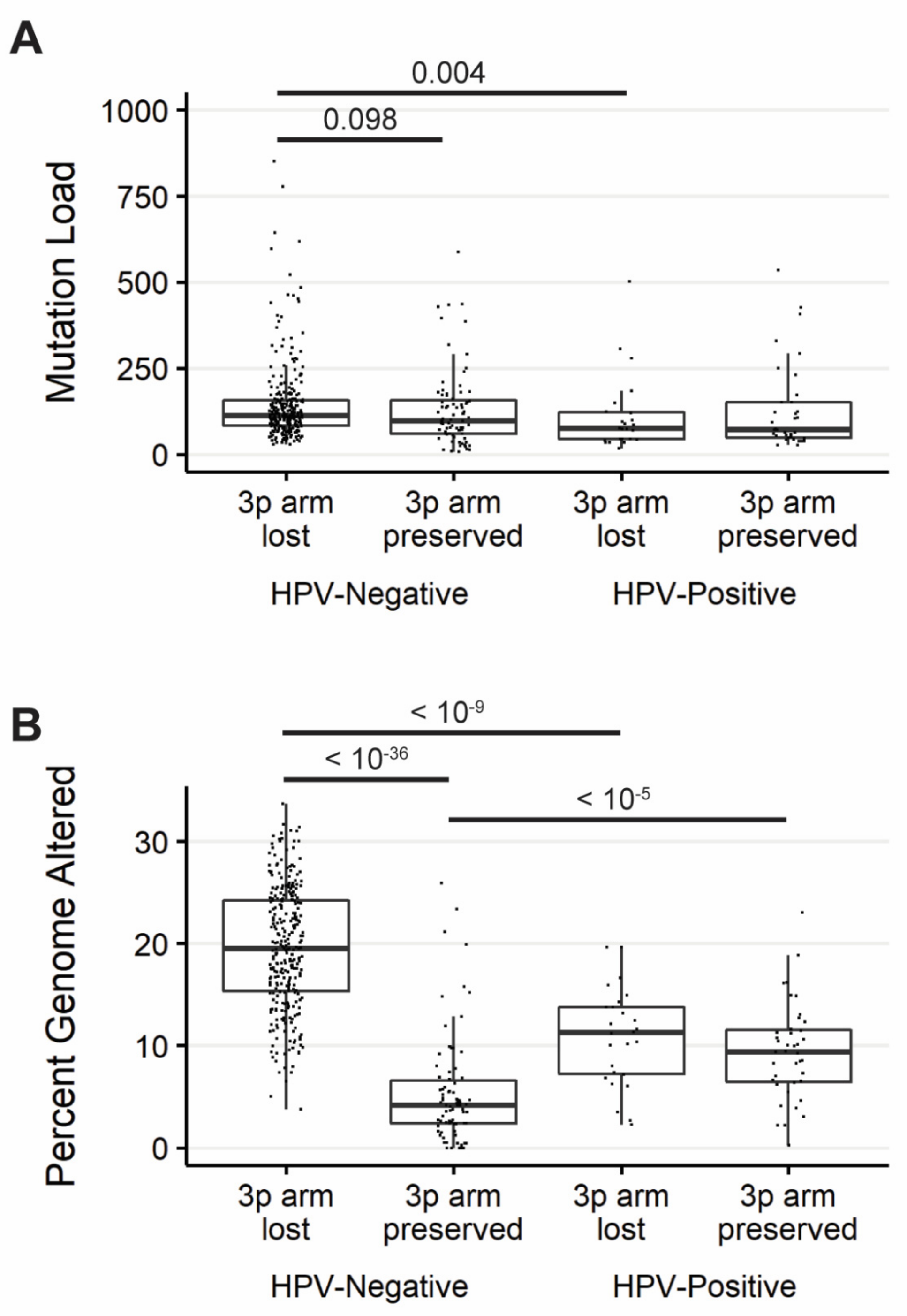

Supplementary Figure S4. Comparison of A) mutation load and B) percent genome altered by 3p arm status and HPV status. Mann-Whitney Wilcoxon tests were used to make the comparisons, and the resulting *p* values are shown.

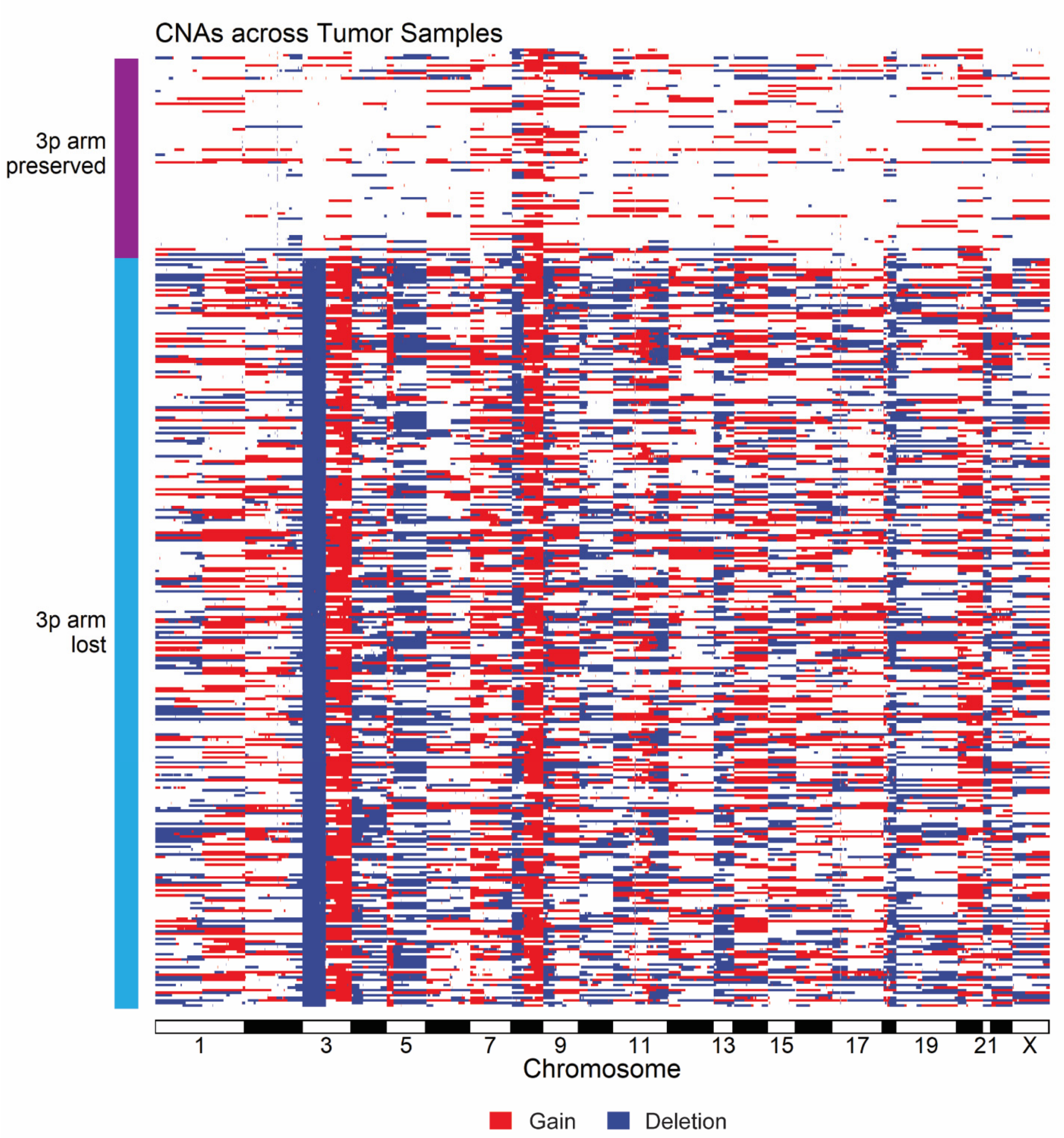

Supplementary Figure S5. Visualization of CNA events across all genes and tumor samples in our HPV-negative cohorts. Every gain and deletion as termed by GISTIC2's CNA analysis in the matrix of 24,776 genes × 375 tumor samples was colored red or blue, respectively.

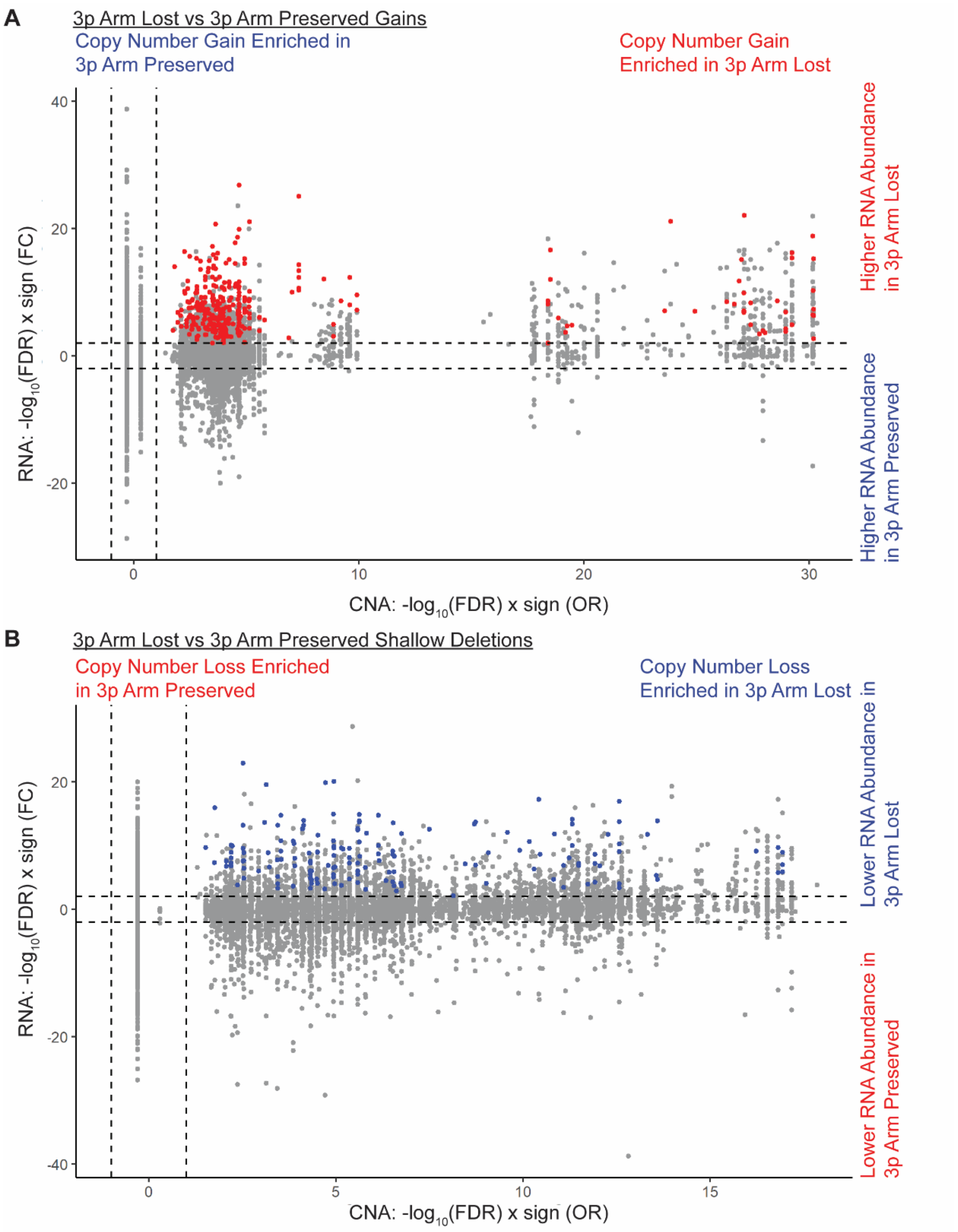

Supplementary Figure S6. Correlation of CNA and mRNA abundance results between by 3p arm status in HPV-negative tumors. A) Deletions and B) Amplifications. Log (base 10) ranked signed FDR values of CNA and mRNA abundance are mapped on the x- and y-axis, respectively. Colored dots represent genes with at least a two-fold change in mRNA abundance. Genes with higher copy number (FDR<0.1) and mRNA abundance (FDR<0.01) in 3p arm loss are colored red, while genes with lower copy number (FDR<0.1) and mRNA abundance (FDR<0.01) in 3p arm loss are colored blue. Genes on the 3p arm are not shown.

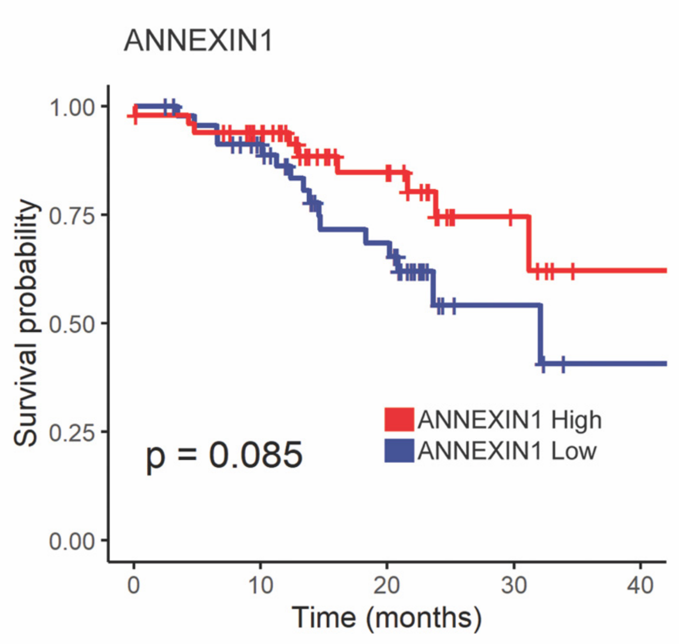

Supplementary Figure S7. Disease-free survival difference by ANNEXIN1 abundance in the CPTAC validation cohort. Patients were grouped by ANNEXIN1 abundance above or below the median. Log-rank test was used for survival comparison.

Supplementary Table S1. Demographic differences by 3p arm status in MSK-IMPACT HNSCC cohort HPV-negative samples.

		HPV-Negative Samples, No. (%) (n=94)		
**Variables**		**3p arm lost (n=68)**	**3p arm preserved (n=26)**	***P* value**
**Age**	Median (range)	58.5 (28-77)	53.5 (20-76)	0.0551
**Sex**	Female	17 (25)	6 (23)	1
	Male	51 (75)	20 (77)	
**Anatomical site**	Hypopharynx	8 (12)	0 (0)	N/A
	Larynx	16 (24)	4 (15)	
	Nasal cavity and paranasal sinuses	3 (4)	3 (12)	
	Nasopharynx	6 (9)	2 (8)	
	Oral cavity	25 (37)	s	
	Oropharynx	10 (15)	0 (0)	
	Unknown primary	0 (0)	0 (0)	
**Smoking history**	Never smoker	23 (34)	11 (42)	0.478
	Current or former smoker	45 (66)	15 (58)	

Supplementary Table S2. Demographic differences by 3p arm status in MSK-IMPACT HNSCC cohort HPV-positive samples.

		HPV-Positive Samples, No. (%) (n=77)		
**Variables**		**3p arm lost (n=30)**	**3p arm preserved (n=47)**	***P* value**
**Age**	Median (range)	58 (31-84)	61 (37-76)	0.755
**Sex**	Female	5 (17)	1 (2)	0.0308
	Male	25 (83)	46 (98)	
**Anatomical site**	Hypopharynx	0 (0)	1 (2)	N/A
	Larynx	1 (3)	1 (2)	
	Nasal cavity and paranasal sinuses	1 (3)	2 (4)	
	Nasopharynx	0 (0)	2 (4)	
	Oral cavity	0 (0)	0 (0)	
	Oropharynx	26 (87)	40 (85)	
	Unknown primary	2 (7)	1 (2)	
**Smoking history**	Never	11 (37)	22 (47)	0.48
	Current or former	19 (63)	25 (53)	

Supplementary Table S3. Demographic differences by fragile site status in all samples.

Variables		All Samples, No. (%) (n=496)		
		**Fragile site lost (n=354)**	**Fragile site preserved (n=142)**	***P* value**
**Age**	Median (range)	60 (19-84)	60 (26-83)	0.29
**Sex**	Female	78 (22)	48 (34)	**0.0085**
	Male	276 (78)	94 (66)	
**Anatomical site**	Oropharynx	42 (12)	37 (26)	**<10^{-5}**
	Hypopharynx	6 (2)	3 (2)	
	Larynx	100 (28)	14 (10)	
	Oral cavity	206 (58)	88 (62)	
**Smoking history**	Non-smoker	64 (23)	47 (39)	**<10^{-4}**
	Light	34 (12)	27 (22)	
	Heavy	176 (64)	46 (38)	
**T category**	T0-T2/TX	143 (41)	68 (53)	**0.030**
	T3-T4	202 (59)	61 (47)	
**N category**	N0-N2a, NX	212 (62)	97 (76)	**0.0045**
	N2b-N3	132 (38)	31 (24)	
**Overall stage**	I-III	114 (37)	57 (49)	**0.027**
	IV	195 (63)	60 (51)	
**Adjuvant radiotherapy**	No	102 (33)	46 (35)	0.74
	Yes	205 (67)	86 (65)	
**HPV status**	Negative	325 (92)	98 (69)	**<10^{-9}**
	Positive	29 (8)	44 (31)	

Supplementary Table S4. Multivariate analysis of overall survival in the HPV-negative cohort by 3p arm status. The multivariate model was constructed through backwards stepwise analysis.

		HPV-negative			
		Univariate		Multivariate	
Variables		HR (95% CI)	*P* value	HR (95% CI)	*P* value
**Fragile site definition**					
**Adjuvant radiotherapy**	Yes *vs.* No	0.736 (0.524 - 1.04)	0.079	0.719 (0.509 - 1.02)	0.062
**Fragile site**	Lost *vs.* Preserved	1.41 (0.959 - 2.07)	0.081	1.49 (0.972 - 2.28)	0.067
**Threshold definition**					
**Adjuvant radiotherapy**	Yes *vs.* No	0.736 (0.524 - 1.04)	0.079	0.751 (0.519 - 1.09)	0.13
**3p arm threshold**	Lost *vs.* Preserved	1.37 (0.898 - 2.1)	0.14	1.48 (0.928 - 2.36)	0.1

Supplementary Tables S5-8: see Excel file

Supplementary Table S9. Pathways significantly overrepresented with SNV and integrated CNA-mRNA differences between 3p arm status. Genes with higher copy number and higher mRNA abundance in 3p arm loss are colored red, genes with lower copy number and lower mRNA abundance in are colored blue, and genes with differences in SNV are uncolored.

1	**Pathway identifier**	R-HSA-198933		
	**Description**	#Entities total: 132<br>Immunoregulatory interactions between a Lymphoid and a non-Lymphoid cell		
	**#Entities mapped in Reactome**	17	**Submitted entities found**	COL2A1, FCGR1A, CD3E, CD3D, CD3G, CRTAM, HCST, LILRA4, KIR2DL1, KIR2DL3, KIR2DL4, KIR3DL1, KIR3DL2, SH2D1A, CD40LG, HLA-A, HLA-B
	***P* value**	$<10^{-7}$	**FDR**	$<10^{-4}$
2	**Pathway identifier**	R-HSA-373076		
	**Description**	#Entities total: 335<br>Class A/1 (Rhodopsin-like receptors)		
	**#Entities mapped in Reactome**	27	**Submitted entities found**	KISS1, CHRM3, POMC, ADRA2B, SST, NPSR1, GAL, TAC3, GALR2, NPB, CNR2, CXCR2, GPBAR1, CXCR6, XCR1, CCR2, CCR5, GNRHR, CXCL9, CXCL10, CXCL11, GPR31, CCL21, CXCR5, GPR18, CXCR3, P2RY10
	***P* value**	$<10^{-6}$	**FDR**	$<10^{-4}$
3	**Pathway identifier**	R-HSA-380108		
	**Description**	#Entities total: 59<br>Chemokine receptors bind chemokines		
	**#Entities mapped in Reactome**	11	**Submitted entities found**	CXCR2, CXCR6, XCR1, CCR2, CCR5, CXCL9, CXCL10, CXCL11, CCL21, CXCR5, CXCR3
	***P* value**	$<10^{-6}$	**FDR**	$<10^{-4}$
4	**Pathway identifier**	R-HSA-375276		
	**Description**	#Entities total: 201<br>Peptide ligand-binding receptors		
	**#Entities mapped in**	19	**Submitted**	KISS1, POMC, SST, NPSR1, GAL, TAC3, GALR2, NPB, CXCR2, CXCR6, XCR1, CCR2, CCR5, CXCL9, CXCL10, CXCL11, CCL21, CXCR5, CXCR3

	**Reactome**		**entities found**	
	***P* value**	$<10^{-5}$	**FDR**	$<10^{-3}$
5	**Pathway identifier**	R-HSA-425366		
	**Description**	#Entities total: 85<br>Transport of bile salts and organic acids, metal ions and amine compounds		
	**#Entities mapped in Reactome**	12	**Submitted entities found**	SLC30A10, SLC30A3, CP, SLC6A3, SLC30A8, SLC22A11, SLC5A11, SLC6A2, SLC13A2, SLC16A8, SLC18A1, SLC6A14
	***P* value**	$<10^{-5}$	**FDR**	$<10^{-3}$
6	**Pathway identifier**	R-HSA-500792		
	**Description**	#Entities total: 468<br>GPCR ligand binding		
	**#Entities mapped in Reactome**	30	**Submitted entities found**	KISS1, WNT9A, CHRM3, POMC, ADRA2B, FZD7, SST, NPSR1, GAL, TAC3, GALR2, GCGR, NPB, CNR2, CXCR2, GPBAR1, CXCR6, XCR1, CCR2, CCR5, GNRHR, CXCL9, CXCL10, CXCL11, GPR31, CCL21, CXCR5, GPR18, CXCR3, P2RY10
	***P* value**	$<10^{-5}$	**FDR**	$<10^{-3}$
7	**Pathway identifier**	R-HSA-112316		
	**Description**	#Entities total: 413<br>Neuronal System		
	**#Entities mapped in Reactome**	25	**Submitted entities found**	KCNH1, KCNS3, KCNG3, NRXN1, LRRTM4, ADCY5, BCHE, KCNMB2, KCNMB3, HTR3E, SLC6A3, KCNV1, ADCY8, PPFIA1, SHANK2, NRXN3, CACNG4, KCNJ16, DLGAP1, CHRNA4, KCNQ2, KCNJ4, PANX2, KCNA3, HRAS
	***P* value**	$<10^{-4}$	**FDR**	0.0065

Supplementary Table S10. TME comparisons by 3p arm status. Scores were computed using the MCP-counter method, normalized using Box-Cox transformation, and compared using linear regression. FDR correction was done using Benjamini-Hochberg method. Significant values are bolded.

**Cell type**	**Fold change [95%CI]**	***P* value**	**FDR**
T cell	0.58 [0.46,0.74]	**$<10^{-5}$**	**$<10^{-4}$**
NK cell	0.68 [0.53,0.86]	**0.0017**	**0.0045**
B cell	0.95 [0.74,1.2]	0.70	0.70
Monocyte	0.63 [0.49,0.80]	**$<10^{-3}$**	**$<10^{-3}$**
Myeloid dendritic cell	0.86 [0.67,1.1]	0.22	0.25
Neutrophil	1.3 [0.98,1.6]	0.069	0.11
Endothelial cell	1.2 [0.95,1.6]	0.12	0.16
Cancer-associated fibroblast	1.4 [1.1,1.8]	**0.010**	**0.020**

Supplementary Table S11. TME comparisons by 3p arm status controlled for PIK3CA gain. Scores were computed using the MCP-counter method, normalized using Box-Cox transformation, and compared using linear regression controlling for PIK3CA gain. FDR correction was done using Benjamini-Hochberg method. Significant values are bolded.

**Cell type**	**Fold change [95%CI]**	***P* value**	**FDR**
T cell	0.68 [0.5,0.93]	**0.016**	**0.064**
NK cell	0.71 [0.51,0.97]	**0.031**	**0.083**
B cell	0.9 [0.65,1.23]	0.50	0.57
Monocyte	0.67 [0.49,0.92]	**0.014**	**0.064**
Myeloid dendritic cell	0.83 [0.6,1.14]	0.24	0.38
Neutrophil	1.03 [0.75,1.42]	0.85	0.85
Endothelial cell	1.16 [0.84,1.59]	0.37	0.50
Cancer-associated fibroblast	1.3 [0.94,1.78]	0.11	0.22

Supplementary Table S12. Comparison of hypoxia profiles by 3p arm status. All eight hypoxia scores in Bhandari *et al.*'s supplementary materials were normalized using Box-Cox transformation and compared using linear regression [17]. FDR correction was done using Benjamini-Hochberg method. All values were significant.

**Score**	**Fold change [95%CI]**	***P* value**	**FDR**
Buffa	1.3 [1.0,1.7]	0.022	0.022
Elvidge	1.4 [1.1,1.8]	0.0044	0.0088
Hu	1.5 [1.2,1.9]	0.0014	0.0038
Ragnum	1.5 [1.2,1.9]	0.0013	0.0038
Seigneuric	1.4 [1.1,1.7]	0.0092	0.012
Sorensen	1.3 [1.0,1.7]	0.020	0.022
West	1.4 [1.1,1.7]	0.0078	0.012
Winter	1.6 [1.3,2.1]	$<10^{-4}$	$<10^{-3}$

Supplementary Table S13. Comparison of hypoxia profiles by 3p arm status controlling for PIK3CA gain. All eight hypoxia scores in Bhandari *et al.*'s supplementary materials were normalized using Box-Cox transformation and compared using linear regression controlling for PIK3CA gain s[17]. FDR correction was done using Benjamini-Hochberg method. Significant values are bolded.

**Score**	**Fold change [95%CI]**	***P* value**	**FDR**
Buffa	1.41 [1.04,1.92]	**0.029**	**0.057**
Elvidge	1.19 [0.87,1.61]	0.27	0.27
Hu	1.53 [1.12,2.08]	**0.0068**	**0.024**
Ragnum	1.33 [0.98,1.81]	0.066	0.11
Seigneuric	1.26 [0.93,1.72]	0.14	0.18
Sorensen	1.21 [0.89,1.65]	0.22	0.25
West	1.51 [1.11,2.05]	**0.0090**	**0.024**
Winter	1.6 [1.18,2.18]	**0.0024**	**0.019**

Supplementary Tables S14-16: Please see excel file.
